# Effect of Nitrogen on Stored-Product Insect Control at Industrial Scale

**DOI:** 10.3390/insects14060518

**Published:** 2023-06-03

**Authors:** Christos I. Rumbos, Maria K. Sakka, Thomas N. Vassilakos, Christos G. Athanassiou

**Affiliations:** Laboratory of Entomology and Agricultural Zoology, Department of Agriculture, Plant Production and Rural Environment, University of Thessaly, 38446 Nea Ionia, Greece; crumbos@uth.gr (C.I.R.); tomvas2001@yahoo.gr (T.N.V.); athanassiou@uth.gr (C.G.A.)

**Keywords:** insect control, modified atmospheres, nitrogen, storage beetles, life stages

## Abstract

**Simple Summary:**

Nitrogen treatment is a promising method for controlling stored product insects. It is based on the alternation of the oxygen level in the atmosphere (lower than 3%), which can be lethal to insects. In the present study, we tested nitrogen treatment (>99%) on a commercial scale to control major stored product insects such as *Sitophilus granarius* (L.), *Sitophilus oryzae* (L.), *Rhyzopertha dominica* (F.), *Prostephanus truncatus* (Horn), *Tribolium confusum* Jacquelin du Val, and *Oryzaephilus surinamensis* (L.). The current work indicates that nitrogen treatment is effective for all life stages and populations tested.

**Abstract:**

In this study, we evaluated the insecticidal effect of nitrogen against *Sitophilus granarius* (L.), *Sitophilus oryzae* (L.), *Rhyzopertha dominica* (F.), *Prostephanus truncatus* (Horn), *Tribolium confusum* Jacquelin du Val, and *Oryzaephilus surinamensis* (L.). Four trials were conducted in chambers containing flour in bags or sacks with >99% nitrogen level. Adults of all the above species, as well as immature life stages (eggs, larvae, and pupae) of *T. confusum* were used in the trials. Our results showed that nitrogen caused high mortality for all species and life stages tested. Some survival was recorded for *R. dominica* and *T. confusum* pupae. Low progeny production was recorded for *S. granarius*, *S. oryzae,* and *R. dominica*. In conclusion, our trials indicated that a high nitrogen environment can provide satisfactory control of various primary and secondary stored-product insect species.

## 1. Introduction

Insect infestation in durable dry commodities can cause extensive qualitative losses and quantitative degradations during the post-harvest stages [1]. Pest management mainly focuses on chemical treatments on different commodities, using fumigants, such as phosphine or other residual insecticides, which are repetitively applied to mitigate post-harvest infestations [2,3]. Moreover, the development of resistance to phosphine and other commonly used insecticides has decreased in various cases with the efficacy of chemical control [4,5,6,7,8,9,10]. Consumers demanding environmentally friendly techniques in food processing and the increase of healthy foods have increased the need to develop new methods and technologies for the control of post-harvest insects. Among these methods, nitrogen is a very promising alternative to traditional chemical control of storage insects [11,12,13]. The use of nitrogen is environmentally friendly, residue-free, and can be used without the need for registration in food storage and processing, based on the current legislation [13,14,15].

The results published so far at both laboratory and field scales are promising and underline the adaptability of this method to different application scenarios, ranging from large silos to chambers [12,13,15,16,17,18,19]. Recently, Agrafioti et al. [19] modeled the way that nitrogen displaces oxygen in pallets with Corinth currants in relation to temperature and treatment interval, while Sakka et al. [13,15] found that the method was effective for a wide range of stored-product insect species in pallets, as long as the percentage of oxygen remains at 1% or lower. Moreover, Navarro et al. [16], in a series of trials in large silos with bulked grains in Cyprus, found that the method caused 100% mortality in secondary pests, but there was some survival in the case of primary pests for the same exposure intervals. Nevertheless, the vast majority of field-scale data produced in the papers indicated above are focused on bulked grains or packaged products in pallets. Still, there are certain gaps in the utilization of the method in other application scenarios, in terms of both storage techniques and types of commodities, that have not been examined in detail yet. For instance, apart from bulked grains or pallets with dried fruits, there is a need to evaluate the use of nitrogen in big bags, which is a common way to store different kinds of commodities. Moreover, given that most of the data available are about grains or dried fruits, it is essential to test nitrogen in different products with lower permeability, such as flour.

Considering the above, and taking into account the need to expand the use of this method in various scenarios, the scope of our study was to add data from industrial applications of nitrogen for the disinfestation of grains that are stored in big bags and also on flour. Therefore, we tested a wide range of target species that include both primary pests, such as the granary weevil, *Sitophilus granarius* (L.) (Coleoptera: Curculionidae), the rice weevil, *Sitophilus oryzae* (L.) (Coleoptera: Curculionidae), the lesser grain borer, *Rhyzopertha dominica* (F.) (Coleoptera: Bostrychidae), and the larger grain borer, *Prostephanus truncatus* (Horn) (Coleoptera: Bostrychidae), and secondary pests, such as the confused flour beetle, *Tribolium confusum* Jacquelin du Val (Coleoptera: Tenebrionidae), and the sawtoothed grain beetle, *Oryzaephilus surinamensis* (L.) (Coleoptera: Silvanidae).

## 2. Materials and Methods

### 2.1. Test Insects

All insect species were maintained at the Laboratory of Entomology and Agricultural Zoology, Department of Agriculture, Crop Production and Rural Environment, University of Thessaly, at 25 °C, 65% relative humidity (r.h.), and continuous darkness. *Sitophilus granarius*, *S. oryzae,* and *R. dominica* individuals were reared on whole wheat kernels, while *P. truncatus* on whole maize kernels. The colonies of *Tribolium confusum* and *O. surinamensis* were reared on wheat flour and oat flakes, respectively. For all species, adult beetles < 1 month old were used in the trials. In the case of *T. confusum*, eggs, larvae, and pupae were also used for experimentation. The eggs were newly hatched (one to two days old), and larvae and pupae were younger than seven days and three days old, respectively. The methods for obtaining the different life stages of *T. confusum* have been described in previous papers [13,15,19].

### 2.2. Experimental Design and Commodity

Four trials were conducted inside a sealed chamber (237.6 m^3^) in which the nitrogen was introduced by using the system that is described in previous works [13,15,19]. For Trials 1 and 2, plastic Petri dishes (9 cm in diameter) were used, while for Trials 3 and 4, we used plastic cylindrical vials (2.5 cm in diameter, 9 cm in height). The vials were penetrated on three different sides (upper, lower, and middle part), and the holes were covered with a U.S. #40 fine mesh screen (0.42 mm openings) to allow air movement in the internal part.

#### 2.2.1. Trials 1 and 2

Trials 1 and 2 were started on 19/4 and 30/4 and were terminated on 27/4 and 8/5, respectively. The day before the nitrogen application, all life stages of *T. confusum* were taken from the rearings and prepared as noted above. Ten individuals from each life stage were placed in each dish, using a separate dish for each life stage. For each location, there were three dishes for each life stage (twelve dishes in total). In each dish, there were small quantities of flour to allow feeding of the exposed individuals. The total number of locations was 13 to represent different sides inside the chamber (156 dishes total). From these, locations 11–13 were placed inside the flour sacks (5 kg each), covered with flour, and the sacks were closed again with sticky tape. A sensor (Centaur Analytics Inc., Ventura, CA, USA) for recording oxygen and nitrogen concentration was placed in each location. A separate series of dishes was placed outside of the treated area and served as control. After the oxygen level reached the desired level, i.e., <1% and nitrogen >99%, the duration of both trials was four days, and the temperature was set at 28 °C during this interval. At the end of the trials, the chamber was opened, and all dishes were transferred to the laboratory to count the surviving individuals. Larvae and adults were counted on the next day, while pupae and eggs were placed for seven days at the rearing conditions mentioned above to evaluate hatching and emergence, respectively. After seven days, the dishes were opened, and all individuals were classified as alive or dead.

#### 2.2.2. Trial 3

This trial was initiated on 19/10 and was terminated on 27/10. The chamber was filled with 36 pallets of soft and hard wheat flour, as well as soft wheat flour with additives. Each pallet contained 13 rows of commercial 5 kg flour sacks. Adults of *T. confusum*, *S. granarius*, *O. surinamensis*, *P. truncatus,* and *R. dominica* were tested in this trial. In the case of *T. confusum*, eggs, larvae, and pupae were also tested, as noted above. One day before the trial, eggs, larvae, pupae, and adults of *T. confusum*, as well as adults of *S. granarius*, *O. surinamensis*, *P. truncatus,* and *R. dominica* were taken from the rearings, and ten individuals from each insect species and life stage were placed in vials, using separate vials for each insect species and life stage. A small amount of food was provided to feed the exposed individuals. Flour was used as a food source in the case of *T. confusum* and *O. surinamensis*, cracked maize kernels in the case of *P. truncatus,* and cracked wheat kernels in the case of *S. granarius* and *R. dominica*.

Vials with insects were placed in 13 locations inside the chamber. Different locations were used inside and outside the flour sacks, under, in the middle, or the upper part of the pallet. The vials placed inside the flour sacks were covered with flour, and the sacks were closed again with sticky tape exactly as before. For each location, there were two vials for each insect and life stage (26 vials in total for each insect species and life stage). Control vials from each insect species and life stage were placed outside the treated area. The temperature inside the nitrogen chamber was recorded with 12 data loggers, which were placed in each test location near the insects, except for one location. Before treatment, two flour samples were collected from each test location. One of the two product samples from each location was stored at the area of the treatment, in a warehouse outside of the chambers, while the other one was kept in the Laboratory of Entomology and Agricultural Zoology in incubators set at 25 °C, 65% r.h., and continuous darkness. Samples were kept for two months and afterward were examined for insects. Sampling was repeated after treatment, and the same procedure of handling and storage was followed.

The duration of the exposure of the insects at <1% O_2_ level was seven days. After the termination of the procedure, the chamber was opened, and all vials were transferred to the laboratory to count the surviving individuals. Counting of the different life stages was carried out as indicated above.

#### 2.2.3. Trial 4

This trial was started on 14/11 and was terminated on 2/12. In this case, the test chamber was filled with 26 big bags with 1000 kg (±10%) of wheat. Adults of *T. confusum*, *S. granarius*, *S. oryzae*, *O. surinamensis*, *P. truncatus,* and *R. dominica*, as well as eggs, larvae, and pupae of *T. confusum* were used in this trial, using the techniques mentioned above, with the small quantities of a food source. Two weeks before the experiment, 2 kg of wheat was placed in glass jars and infested with adults of *S. granarius*, *S. oryzae,* and *R. dominica*, with separate jars for each insect species, and kept at 25 °C, 65% r.h., and continuous darkness. After two weeks, all adults were removed, and the infested grains, including eggs and various larval stages of all three insect species, were used for experimentation. Namely, 18 g of the infested grains was placed in plastic cylindrical vials. Then, vials with *S. granarius*, *S. oryzae,* and *R. dominica* adults, as well as grains infested with *S. granarius*, *S. oryzae,* and *R. dominica* were placed in 14 locations in the test chamber. The vials were placed inside (approximately at a depth of 30 cm) and outside of the wheat (on the surface of the big bags), as well as under the pallets used as a base for the big bags. For each location, there were two vials from each insect species and life stage (28 vials in total for each insect species and life stage). Moreover, vials with adults of *T. confusum*, *O. surinamensis,* and *P. truncatus*, as well as eggs, larvae, and pupae of *T. confusum* were placed in six locations within the test chamber. A separate series of three vials from each insect species and life stage was placed outside the treated area and served as a control. The temperature in the test chamber was recorded with six data loggers, which were placed in selected locations. In this trial, the insects were exposed for 19 days at <1% O_2_. Sampling of wheat was done before and after treatment, as described above. After the termination of the procedure, the chamber was opened, and all vials were evaluated, as in the case of Trial 3.

### 2.3. Data Analysis

For Trials 1 and 2, all data, separately for each Trial, were submitted to a One-Way Analysis of Variance to indicate if there were differences among trials. For Trials 3 and 4, the data, separately for each trial, insect species, and life stage, were submitted to a One-Way Analysis of Variance (ANOVA) to indicate if there were differences among treatments. In all cases, means were separated using the Tukey–Kramer HSD test at 0.05% [20].

## 3. Results

### 3.1. Trials 1 and 2

Regarding both trials, there were significant differences in mortality levels among trials when the control dishes were included in the analysis (Table 1). Nevertheless, in all cases, insect mortality of the exposed individuals was 100% (Table 2). Regarding the control mortality, we recorded negligible mortality of the untreated adults, but more than one-half of the unexposed eggs were dead.

### 3.2. Trial 3

Significant differences were noted in all cases between treatment and control (Table 3). Complete control (100%) was achieved in all cases where nitrogen was applied, with the exception of *T. confusum* pupae and adults and *R. dominica* adults, for which 92.0, 99.6, and 92.7% mortality was recorded, respectively (Table 4). In contrast, control survival was high and ranged between 49.6 and 100% (Table 4). No significant differences were recorded among the different locations that were treated with nitrogen, given that in most cases, mean mortality was 100% (Table 4). No live insects were found in the flour samples collected before and after treatment and stored either in the test area or in the Laboratory of Entomology and Agricultural Zoology.

### 3.3. Trial 4

Complete (100%) control was achieved against all life stages of *T. confusum*, as well as against adults of *S. granarius*, *S. oryzae*, *R. dominica*, *O. surinamensis,* and *P. truncatus* with the exception of *T. confusum* pupae (Table 4). Similar to Trial 3, no significant differences were noted among different locations that were treated with nitrogen, given that in most cases, mean mortality was 100% (Table 4). Regarding progeny production numbers in the vials that contained infested grains, significant differences were recorded among treatments (nitrogen application, control) in all cases, with the exception of dead *R. dominica* individuals (Table 5). Progeny production in the vials with grains infested by *S. granarius*, *S. oryzae,* and *R. dominica* is shown in Table 6. No live insects were detected in the wheat samples collected after treatment and stored either in the test area or the laboratory. In contrast, alive individuals of *S. oryzae*, *R. dominica*, *T. confusum,* and the rusty grain beetle, *Cryptolestes ferrugineus* (Stephens) (Coleoptera: Laemophloeidae), a species that had not been included in the trials, were detected in the wheat samples collected before treatment.

## 4. Discussion

Based on the information collected from all four trials, we can conclude that the application of nitrogen is very effective against the different species and life stages tested here. Control was complete even with eggs and pupae of *T. confusum*, which is generally considered a tolerant life stage to the stress caused by changes to oxygen levels [12,17,18,21]. Moreover, nitrogen killed almost all insects that had been placed inside the commodity, indicating that the replacement of air by nitrogen is progressing in a uniform way in the entire mass of the treated commodity. This observation is in agreement with the data that are reported by Agrafioti et al. [19] for the distribution of oxygen within similar chambers, underlying that this distribution is not affected much by the prevailing temperatures. The findings of the present study are particularly important in the case of flour, where airflow is much more limited as compared with bulked grains that have large interstitial air zones among kernels. Similar results for different exposure intervals have been illustrated by other researchers, as well as with different experimental protocols [11,13,15,16,17,19,22,23,24]. These results underline the merit of experimentation at the industrial/commercial scale in order to obtain standardized application conditions that can be adopted in different types of commodities, facilities, and conditions.

Our results illustrate the inferences necessary for an effective nitrogen application under the system used here. In principle, we have found that primary insect pests were much more tolerant to nitrogen than secondary ones, which is, to a certain degree, expected as the immature development of these species occurs within the grain kernels. Moreover, immature stages of certain species may be more tolerant to controlled or modified atmospheres than adults [12,15,17,25]. The same holds in the case of gases, where eggs are usually the most tolerant life stage [26,27,28,29]. For instance, Gourgouta et al. [28] found that complete control of eggs of the khapra beetle, *Trogoderma granarium* (Everts) (Coleoptera: Dermestidae), required 1000 ppm of phosphine for three days, while the respective dose for the other life stages, e.g., eggs or diapausing larvae were controlled at 50 ppm. For sulfuryl fluoride, Athanassiou et al. [27] found that stored-product psocid eggs could survive concentrations lethal for the rest of their life stages, i.e., nymphs and adults. In Trial 1, based on the short protocol, different life stages of *T. confusum* were dead in all locations, which means that the effect of nitrogen is not location specific. Although the distribution of nitrogen within the treated area seemed uniform, there might have been some “oxygen nests”, especially at the beginning of the trial, which may have allowed some insect survival through shorter exposures in some locations, as compared with others. Previous studies have shown that stored-product insects can survive for long intervals, even when the oxygen level is as low as 3% [11,16,30,31]. Nevertheless, survival in these locations did not affect the overall efficacy of the trials or mortality of the different life stages of the insects tested.

We consider that the utilization of vials with already-infested commodities can be the ultimate “crash test” to test this method. This is due to the fact that the sole use of a predefined number of individuals that have been taken from laboratory cultures may result in a certain bias, given that these individuals might have been stressed during their handling and transportation. In contrast, allowing adults to oviposit for a given interval to grain kernels will result in a large (but unknown) number of eggs and young larvae closer to their natural environment. If, after the termination of a given trial, these vials do not contain alive individuals, the vials should still be kept in an incubation chamber with desirable conditions, as this procedure may reveal a level of survival some weeks later. In our case, this is where we have found most of the survival, suggesting that the exposure intervals should be longer. Vials with already-infested grains have been proven useful in different types of trials at a commercial scale, such as in heat treatments [32], phosphine application [33,34,35], and also in modified atmospheres [15,17,19]. This technique suggested that, in contrast with *T. confusum*, our tests showed that the “short protocol” was unable to provide 100% kill of *R. dominica* adults. Hence, a “species-specific” protocol may be more applicable in nitrogen chambers, as compared with a “commodity-specific” protocol.

Some adults were able to emerge from the nitrogen-treated infested grains, but this number was minimal (0.6, 1.4, and 0.3 per vial for *S. granarius*, *S. oryzae,* and *R. dominica*, respectively) and considerably lower than the respective adult emergence in the controls, which was extremely high per sample unit. At the same time, the grains sampled were also found to be naturally infested by *C. ferrugineus*, and thus, some conclusions can be drawn for the effect of nitrogen on this species as well. As during sampling or after the removal of the parental adults in the vials that contained infested grains, there was no visible sign of live insect individuals; we assume that the adults that were recorded at the end of the incubation period were due to the egg survival after the application. Still, our results show that this survival did not follow any specific pattern that confirms the uniform distribution of nitrogen within the chamber. Similar findings have been reported in previous trials with nitrogen chambers [13,15,17].

## 5. Conclusions

In summary, the application of nitrogen through the currently used protocols proved very effective for the range of species tested here and can sufficiently protect flour and wheat from infestation, even if 100% insect mortality was not achieved in all trials. Moreover, one of the key parameters that affect the efficacy of the method is temperature, which was effectively monitored by the remote sensors tested. In addition, the duration of the treatment can be further shortened through the increase of the temperature level within the chamber, as suggested by Athanassiou et al. [17], who examined the effect of a wide temperature range in nitrogen chambers for the control of *T. confusum*, *O. surinamensis*, and the tobacco moth, *Ephestia elutella* (Hübner) (Lepidoptera: Pyralidae). The emergence of the primary pests on the wheat samples was extremely low and consisted of an additional indication that the system is effective, and we estimate that this emergence level would have been higher in the case of the application of fumigants, such as phosphine. Finally, we consider that for specific hard-to-kill insects, such as *R. dominica*, the adoption of a longer protocol may be necessary.

## Figures and Tables

**Table 1:** ANOVA parameters for mortality of different *Tribolium confusum* eggs, larvae, pupae, and adults exposed to high nitrogen environment.

	*df*	*Tribolium confusum*							
		Adults		Larvae		Pupae		Eggs	
		*F*	*p*	*F*	*p*	*F*	*p*	*F*	*p*
Trial 1	1	15618.6	<0.01	9824.3	<0.01	668.6	<0.01	3138.6	<0.01
Trial 2	1	3640.0	<0.01	571.4	<0.01	4754.3	<0.01	3138.6	<0.01

**Table 2:** Mean mortality (% ±SE) of *Tribolium confusum* eggs, larvae, pupae, and adults exposed to high nitrogen environment.

		*Tribolium confusum*			
		Adults	Larvae	Pupae	Eggs
Trial 1	Control	3.3 ± 0.7	23.3 ± 0.7	40.0 ± 2.2	56.7 ± 0.7
	Nitrogen	100.0 ± 0.0	100.0 ± 0.0	100.0 ± 0.0	100.0 ± 0.0
Trial 2	Control	6.7 ± 1.5	33.3 ± 1.5	46.7 ± 0.7	56.7 ± 0.7
	Nitrogen	100.0 ± 0.0	100.0 ± 0.0	100.0 ± 0.0	100.0 ± 0.0

**Table 3:** ANOVA parameters for mortality of *Tribolium confusum*, *Sitophilus granarius*, *Oryzaephilus surinamensis*, *Rhyzopertha dominica*, *Prostephanus truncatus,* and *Sitophilus oryzae* adults, as well as *T. confusum* eggs, larvae, and pupae exposed to high nitrogen in two trials (*df* = 1).

		*T. confusum*				*S. granarius*	*O. surinamensis*	*R. dominica*	*P. truncatus*	*S. oryzae*
		Eggs	Larvae	Pupae	Adults	Adults	Adults	Adults	Adults	Adults
Trial 3	*df*	1	1	1	1	1	1	1	1	n.t.
	*F*	1307.2	442.6	25.0	4134.7	10,179	2941.1	194.7	3 × 10^16^	
	*p*	<0.001	<0.001	<0.001	<0.001	<0.001	<0.001	<0.001	<0.001	
Trial 4	*F*	10	135.9	5.9	3 × 10^16^	115.6	878.8	5.2	301.0	998.4
	*p*	0.007	<0.001	0.03	<0.001	<0.001	<0.001	0.04	<0.001	<0.001

n.t.: not tested.

**Table 4:** Mean mortality (±SEM) of *Tribolium confusum*, *Sitophilus granarius*, *Oryzaephilus surinamensis*, *Rhyzopertha dominica*, *Prostephanus truncatus,* and *Sitophilus oryzae* adults, as well as *T. confusum* eggs, larvae, and pupae exposed to high nitrogen environment.

		*T. confusum*				*S. granarius*	*O. surinamensis*	*R. dominica*	*P. truncatus*	*S. oryzae*
		Eggs	Larvae	Pupae	Adults	Adults	Adults	Adults	Adults	Adults
Trial 3	Control	40.0 ± 5.8 B	46.7 ± 8.8 B	50.4 ± 19.9 B (49.6 ± 19.9 B) *	3.3 ± 3.3 B	3.3 ± 3.3 B	10.0 ± 5.8 B	3.3 ± 3.3 B	0.0 ± 0.0 B	n.t.
	Nitrogen	100.0 ± 0.0 A	100.0 ± 0.0 A	92.0 ± 2.0 A (8.0 ± 2.0 A) *	99.6 ± 0.4 A	100.0 ± 0.0 A	100.0 ± 0.0 A	92.7 ± 2.1 A	100.0 ± 0.0 A	n.t.
Trial 4	Control	83.3 ± 12.0 b	60.7 ± 7.7 b	60.4 ± 10.1 b (33.2 ± 12.6) *	0.0 ± 0.0 b	43.3 ± 12.0 b	13.3 ± 6.7 b	90.0 ± 10.0 b	45.8 ± 7.1 b	20.0 ± 5.8 b
	Nitrogen	100.0 ± 0.0 a	100.0 ± 0.0 a	84.0 ± 4.2 a (16.0 ± 4.2) *	100.0 ± 0.0 a	100.0 ± 0.0 a	100.0 ± 0.0 a	100.0 ± 0.0 a	100.0 ± 0.0 a	100.0 ± 0.0 a

Within each trial and column, means followed by the same upper—(Trial 3) or lower-case letter (Trial 4) do not differ significantly. Where no letters exist, no significant differences were noted (HSD test at *p* = 0.05); n.t.: not tested; * Percentage of adult emergence.

**Table 5:** ANOVA parameters for progeny counts of *Sitophilus granarius*, *Sitophilus oryzae,* and *Rhyzopertha dominica* from infested grains exposed to a high nitrogen environment in Trial 4.

		*S. granarius*		*S. oryzae*		*R. dominica*	
	*df*	Alive	Dead	Alive	Dead	Alive	Dead
*F*	1	16.5	15.3	63.5	31.8	98.2	0.032
*p*	1	<0.001	<0.001	<0.001	<0.001	<0.001	0.859

**Table 6:** Progeny counts (±SE) of *Sitophilus granarius, Sitophilus oryzae,* and *Rhyzopertha dominica* from infested grains exposed to a high nitrogen environment in Trial 4.

	*S. granarius*		*S. oryzae*		*R. dominica*	
	Alive	Dead	Alive	Dead	Alive	Dead
Control	4.3 ± 2.6 a	29.0 ± 7.9 a	74.7 ± 32.9 a	56.3 ± 24.9 a	5.3 ± 1.3 a	23.7 ± 0.7
Treated	0.6 ± 0.2 b	14.4 ± 1.0 b	1.4 ± 0.4 b	12.8 ± 1.1 b	0.3 ± 0.1 b	25.2 ± 2.8

Within each column, means followed by the same lower-case letter do not differ significantly. Where no letters exist, no significant differences were noted (HSD test at *p* = 0.05).

## Data Availability

Data will be made available upon request.
